# Regional Variation in Rates of Total Knee Arthroplasty Among Medicare Beneficiaries

**DOI:** 10.1001/jamanetworkopen.2020.3717

**Published:** 2020-04-28

**Authors:** Michael M. Ward, Abhijit Dasgupta

**Affiliations:** 1 National Institute of Arthritis and Musculoskeletal and Skin Diseases, National Institutes of Health, Bethesda, Maryland

## Abstract

**Importance:**

Rates of total knee arthroplasty vary widely across the United States. Whether this variation is associated with differences in patient characteristics or physician practice is unknown.

**Objectives:**

To determine regional variations in rates of total knee arthroplasty after accounting for the prevalence of knee arthritis and other potentially associated patient risk factors and to assess the correlation of these variations with measures of access to care and surgical indications.

**Design, Setting, and Participants:**

This retrospective national cohort study used Medicare data on more than 24 million deidentified beneficiaries annually from 2011 to 2015. Individuals included had fee-for-service coverage, were 65 to 89 years of age, and resided in 1 of 306 health referral regions. Data were analyzed from September 13, 2018, to August 15, 2019.

**Main Outcomes and Measures:**

Rate of primary total knee arthroplasty indexed to the national rate using observed to expected ratios. The expected numbers of arthroplasty procedures were derived from estimates based on beneficiaries’ demographic and clinical characteristics. Observed to expected ratios were confounded by race/ethnicity; thus race/ethnicity–stratified analyses were conducted.

**Results:**

In 2011, there were 218 282 total knee arthroplasty procedures among 24 583 706 white Medicare beneficiaries (mean [SD] age 74.2 [6.9] years; 54.6% women). The rate of arthroplasty during the study period (5 years) was 9.3 per 1000 person-years. Adjustment for clinical characteristics reduced the spread in observed to expected ratios among regions by 29% compared with adjustment for age and sex alone. However, substantial variation remained, with observed to expected ratios that ranged from 0.61 in Newark, New Jersey, to 1.82 in Idaho Falls, Idaho. High ratios were primarily present in the upper Midwest, Great Plains, and Mountain West regions. Higher ratios were associated with regions where beneficiaries had fewer outpatient visits (Spearman correlation [*r*], −0.64; 95% CI, −0.70 to −0.56) and with regions having more surgeons per capita who performed knee arthroplasty (*r* = 0.27; 95% CI, 0.16-0.37). Higher ratios were associated with higher rates of arthroplasty procedures among beneficiaries with dementia (*r* = 0.36; 95% CI, 0.25-0.46), peripheral vascular disease (*r* = 0.52; 95% CI, 0.42-0.61), and skin ulcers (*r* = 0.43; 95% CI, 0.32-0.53), which are relative contraindications to arthroplasty.

**Conclusions and Relevance:**

Substantial regional variation in rates of total knee arthroplasty remained after adjustment for patient characteristics. Coexistence of high observed to expected ratios and high rates among patients at greater surgical risk suggested overuse of knee arthroplasty in some regions.

## Key Points

**Question:**

How variable are rates of total knee arthroplasty across the United States after accounting for the prevalence of knee arthritis and other patient risk factors?

**Findings:**

In this cohort study of more than 24 million Medicare beneficiaries annually from 2011 to 2015, observed to expected ratios for total knee arthroplasty ranged from 0.61 in Newark, New Jersey, to 1.82 in Idaho Falls, Idaho, suggesting areas of relative underuse and overuse. Regions with higher than expected rates were also associated with high rates among patients having relative contraindications to knee arthroplasty.

**Meaning:**

Decision-making thresholds for performing total knee arthroplasty appear to differ across the US in a pattern suggesting overuse in some regions.

## Teaser

This nationwide cohort study assesses whether rates of total knee arthroplasty are associated with the prevalence of knee arthritis, other potential risk factors, or geographic region among Medicare beneficiaries 65 to 89 years of age.

## Introduction

Total knee arthroplasty (TKA) is a commonly used and highly effective treatment for patients with chronic knee pain and functional limitations, most often due to osteoarthritis, not responsive to conservative interventions.^1,2,3,4,5^ Rates of TKA vary widely across the United States.^6^ Rates in some Midwestern and Western states have been reported to be 4 times as high as in other US regions, raising questions about whether TKA is either overused or underused in different regions.^7,8,9,10,11^ Previous studies have not controlled for factors other than patient age, sex, and race/ethnicity, leaving open the possibility that differences in the prevalence of knee osteoarthritis contribute to variations in TKA rates. Physician practice patterns have been implicated in regional variations for other surgical procedures and may have a role in shaping patient preferences for TKA.^12,13,14,15,16^

In the present study, we examined rates of primary TKA among Medicare beneficiaries to determine if US regional differences were present after accounting for patient characteristics associated with knee arthritis or comorbidities. To assess physician practice patterns, we examined whether the characteristics of patients who received TKA varied in association with regions. To evaluate potential overuse or underuse, we compared regions with the national mean rate and examined use among patients with relative contraindications to TKA.

## Methods

### Data Source

In this retrospective cohort study, we examined primary TKA among Medicare beneficiaries using 100% Medicare Part A and Part B fee-for-service claims data from 2011 to 2015. We used 5 years of data to provide stable estimates. Deidentified data were made available by the Centers for Medicare and Medicaid Services (CMS) through a data use agreement. This study followed the Strengthening the Reporting of Observational Studies in Epidemiology (STROBE) reporting guideline for cohort studies. The study protocol was approved by the National Institute of Diabetes and Digestive and Kidney Diseases institutional review board, which waived the requirement for obtaining informed consent because patient data were deidentified. Data were analyzed from September 13, 2018, to August 15, 2019.

### Inclusion Criteria and Measures

We included all Medicare beneficiaries aged 65 to 89 years who lived in 1 of the 50 states or the District of Columbia, were enrolled in Medicare Parts A and B, and were not enrolled in Medicare managed care plans. We excluded persons aged 90 years or older because this group is overrepresented in the Midwest. Rates of primary TKA are low in persons aged 90 years or older.^7^ Follow-up ended when the beneficiary reached 90 years of age or at the time of death or enrollment in managed care.

Among these beneficiaries, we identified all primary TKA procedures in the hospitalization files based on corresponding procedural codes (*International Classification of Diseases, Ninth Revision, Clinical Modification* [*ICD-9-CM*] code 81.54; *International Statistical Classification of Diseases, Tenth Revision, Clinical Modification* [*ICD-10-CM*] code 0SRC or 0SRD). During the study years, TKA among Medicare beneficiaries was largely performed as an inpatient procedure.^17^ We tabulated primary TKA procedures by hospital referral region (HRR) as defined by *The Dartmouth Atlas of Health Care*.^11^ The HRRs are 306 tertiary medical care regions, labeled by their largest city, that reflect local referral patterns for major surgical procedures. Each HRR has a minimum population of 120 000. We used linkages provided by the CMS to map each beneficiary’s zip code of residence to 1 of 306 HRRs.^18^

We abstracted data on patient factors potentially associated with the likelihood of TKA. Demographic characteristics included age, sex, and race/ethnicity (white, black, Hispanic, Asian, and other). We analyzed race/ethnicity because individuals from minority races/ethnicities are less likely than white individuals to undergo TKA.^9,10^ We identified beneficiaries as having knee osteoarthritis (*ICD-9-CM* code 715.X6; *ICD-10-CM* code M17) or knee symptoms (*ICD-9-CM* code 719.X6; *ICD-10-CM* code M2506, M2526, M2536, M2546, M2556, M2566, M2576, or M2586) based on outpatient visit diagnosis codes. Because knee osteoarthritis may be underrecorded in claims, we also included 3 area-level measures of knee osteoarthritis risk factors: obesity, smoking, and occupational physical activity.^19^ We used county-level data from the 2011 Behavioral Risk Factor Surveillance System and county-to-HRR crosswalks to estimate the prevalence of obesity and smoking in each HRR.^20,21,22^ Similarly, we used county-level data from the 2011 to 2015 US Census files to estimate the proportion of adults engaged in physically demanding occupations (ie, construction, installation, building maintenance, firefighting, and farming) in each HRR.^23^

Because comorbidities may affect considerations for TKA, we identified the presence of 20 comorbid conditions, based on inpatient and outpatient claims updated annually, using the CMS Chronic Conditions Warehouse (eTable 1 in the Supplement).^24^ Total knee arthroplasty is less common among persons of low socioeconomic status.^25^ We categorized beneficiaries as having low socioeconomic status if they received government subsidies for medical insurance premiums. In addition, we computed an area-based measure of socioeconomic status, derived from 7 economic characteristics of the zip code of residence from the 2010 Census.^26^

We examined 5 measures potentially associated with access to TKA. We classified beneficiaries as living in a rural area if their zip code was outside an urban center or cluster in the 2010 Census.^20,27^ We used the annual number of visits for chief concerns of the knee to any clinician and visits for any reason as measures of access to outpatient care. Because local market penetration by managed care plans can influence the care of patients in fee-for-service plans, we used CMS data on the proportion of Medicare beneficiaries enrolled in managed care plans in each county and year to compute Medicare managed care penetration in each HRR.^28,29^ We also counted the number of surgeons in each HRR who performed primary TKA among Medicare beneficiaries in the study years, based on the National Provider Identifier of the operating surgeon. We divided this number by the Medicare population of each HRR to measure TKA surgeon density. We used this measure, rather than the number of orthopedic surgeons, because approximately one-half of US orthopedic surgeons perform arthroplasty procedures.^30^

### Statistical Analysis

We used a relative metric that compared rates of primary TKA among HRRs with the national mean, which facilitated identification of possible overuse and underuse. We first computed the expected number of TKA procedures in each HRR based on models estimated using all beneficiaries across the nation. We then compared the observed number of TKA procedures in each HRR to the expected number as an observed to expected ratio (OER).

To derive the expected number of TKA procedures, we used Poisson models to assess each beneficiary’s probability of TKA, based on their covariates. In the initial model, we included only age (5 categories: 65-69, 70-74, 75-79, 80-84, and 85-89 years), sex, race/ethnicity, and age-sex interaction as covariates. In the full model, we also included indicator variables for knee osteoarthritis or symptoms, 20 comorbid conditions, and classification as low socioeconomic status as well as area-level measures of obesity, smoking, physically demanding occupations, and socioeconomic status. We specified separate models for each year. We summed the probabilities of TKA of beneficiaries in an HRR in each year to derive the expected number of TKA procedres and summed these for the 5 years. We then divided the observed number by the expected number to obtain the OER in each HRR. An OER of 1.0 would indicate an HRR with an observed rate at the national mean expected rate. We used this approach rather than a hierarchical linear model because it was computationally easier with a very large sample and provided the same results.

To determine whether differences in clinical characteristics were associated with differences in OERs among HRRs, we compared the distribution of OERs based on the full model to OERs based on the demographics-only model. We also examined associations between OERs based on the full model and 5 measures of access to care across HRRs: percentage of beneficiaries that lived in rural areas, annual number of outpatient visits for any reason and visits for knee concerns, percentage of beneficiaries in managed care plans, and density of TKA surgeons. Confidence intervals (CIs) for Spearman correlations (*r*) were based on 5000 bootstrap samples.

To examine physician practice patterns, we performed 2 analyses to investigate whether the characteristics of beneficiaries who received TKA differed among HRRs in association with high or low OERs. First, we computed OERs in 4 strata based on quartiles of expected probability of TKA, ranging from very low probability to highest probability. This analysis provided an assessment of how stable OERs were across beneficiary subgroups with different estimated probabilities of TKA. Second, we computed rates of TKA among beneficiaries with either dementia, peripheral vascular disease, or chronic skin ulcers, which are relative contraindications to TKA, and correlated these rates with OERs.^31^ Positive correlations were considered to indicate overuse in high OER regions. Similarly, we computed rates of TKA among beneficiaries with congestive heart failure, depression, or diabetes, which are associated with poorer TKA outcomes, and correlated these with OERs.^32,33^ We also correlated OERs with the rate of TKA among beneficiaries aged 65 to 69 years who had no comorbidity. In validation studies, Medicare claims for dementia had a positive predictive value (PPV) of 83% and depression had a PPV of 66%, while claims for peripheral vascular disease, congestive heart failure, and diabetes had PPVs of more than 92%.^34,35,36,37^ Claims for leg ulcers among patients with diabetes had a PPV of 81%.^38^

Finally, we examined the number of TKA procedures per surgeon across HRRs, to assess whether rates were associated with a few high-volume surgeons. We used SAS, version 9.4 (SAS Institute Inc) for all analyses.

## Results

In 2011, there were 239 950 primary TKA procedures among 28 808 011 beneficiaries, whereas in 2015, there were 262 013 primary TKA procedures among 30 177 710 beneficiaries. The OERs based on adjustment for age, sex, and race/ethnicity ranged from 0.52 (Bronx, New York) to 1.72 (Idaho Falls, Idaho) (eFigure 1 in the Supplement). The HRRs with the highest OERs had predominantly white populations, whereas the HRRs with the lowest OERs had large ethnic minority populations (eTable 2 in the Supplement). Despite adjustment for race/ethnicity, correlations remained between OERs and the racial composition of HRRs, indicating residual confounding (eFigure 2 in the Supplement). Therefore, we used race/ethnicity–specific models in subsequent analyses. Because white individuals comprised 84.6% of the sample, our analyses focused on associations among white beneficiaries. There were 218 282 primary TKA procedures among 24 583 706 white beneficiaries in 2011 (mean [SD] age 74.2 [6.9] years; 54.6% women), and 236 054 TKA procedures among 25 341 532 white beneficiaries in 2015. The rate of arthroplasty during the study period (5 years) was 9.3 per 1000 person-years.

**Figure 1.**
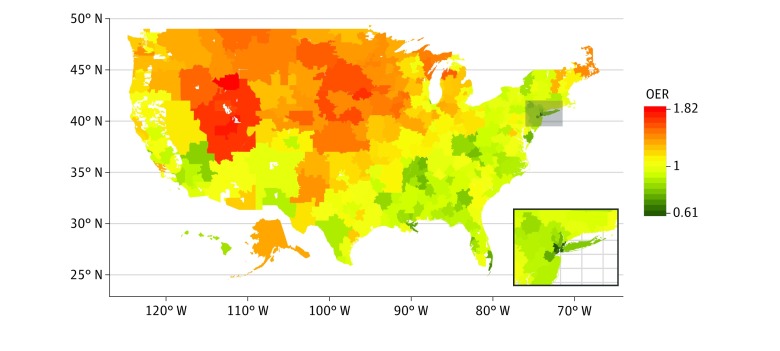
Observed to Expected Ratios (OERs) for Rates of Primary Total Knee Arthroplasty Among White Medicare Beneficiaries by Health Referral Region Ratios greater than 1.0 indicate higher than expected rates of total knee arthroplasty, while ratios less than 1.0 indicate lower than expected rates. Insert shows the New York City region.

The clinical characteristics of white beneficiaries varied widely among HRRs. For example, the percentage of beneficiaries with low socioeconomic status ranged from 2.1% to 35.6%, and the percentage with visits for knee symptoms ranged from 3.3% to 9.6% (eTable 3 in the Supplement). Compared with OERs adjusted for demographic characteristics only, further adjustment for differences in the prevalence of knee osteoarthritis and its risk factors, comorbidities, and socioeconomic status narrowed the spread among OERs by 29% (eFigure 3 and eTable 4 in the Supplement). However, substantial variation remained, with OERs ranging from 0.61 (Newark, New Jersey) to 1.82 (Idaho Falls, Idaho). Regions with high OERs were concentrated in the upper Midwest, Great Plains, and Mountain West, whereas HRRs in the New York City region, south Florida, Chicago, and the New Orleans and Memphis areas had low OERs (Figure 1; eTable 5 in the Supplement). The OERs varied little during the 5 years (eFigure 4 in the Supplement).

**Figure 2.**
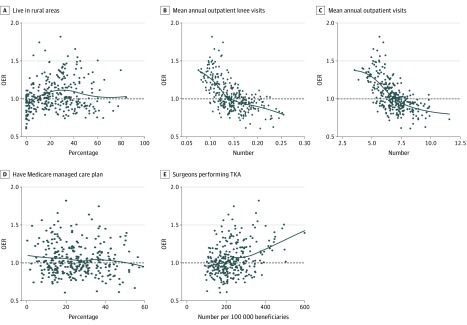
Associations Between Observed to Expected Ratios (OERs) for Rates of Primary Total Knee Arthroplasty (TKA) and Measures of Access to Care Among Health Referral Regions Access to care measures included (A) the percentage of beneficiaries living in rural areas, (B) the mean annual number of outpatient visits for knee concerns or (C) any outpatient visit, (D) the percentage of beneficiaries in Medicare managed care plans, and (E) the number of surgeons performing TKA procedures per 100 000 beneficiaries. Each black dot represents a health referral region; black lines, locally estimated scatterplot smoothing curves of the association.

The HRRs whose residents had fewer outpatient visits for knee concerns (*r* = −0.63; 95% CI, −0.69 to −0.55) or fewer visits for any reason (*r* = −0.64; 95% CI, −0.70 to −0.56) were associated with higher OERs than regions whose residents had more outpatient visits (Figure 2). The association between OERs and percentage of rural residents was weaker (*r* = 0.15; 95% CI, 0.03-0.27), and no association was found between OERs and percentage with managed care (*r* = −0.07; 95% CI, −0.18 to 0.05). The HRRs with more TKA surgeons per capita were associated with higher OERs than those with fewer surgeons (*r* = 0.27; 95% CI, 0.16-0.37).

The high OERs in many Western states and the association with outpatient visit frequency suggested that limited geographic access to care may contribute to the association with high OERs. To test this possibility, we computed OERs for the urban centers and outlying areas of 9 HRRs (3 with high OERs, 3 with OERs close to 1.0, and 3 with low OERs) (Table). The OERs of outlying areas were higher than those of the urban centers in each region except Lexington, Kentucky, suggesting greater use of TKA among residents of outlying areas. However, OERs in the urban centers of Salt Lake City, Utah; Lincoln, Nebraska; and Wichita, Kansas were each greater than 1.1, indicating higher than expected rates in these cities.

**Table.** Observed to Expected Ratios for Primary Total Knee Arthroplasty Rates Among 1 277 714 White Medicare Beneficiaries in the Urban Centers or Outlying Areas of Selected Health Referral Regions

Health referral region^a^	Beneficiaries living in the urban center, %	Observed to expected ratio			
		Overall	Urban center	Outlying area	Difference, outlying minus urban
Lexington, Kentucky	14.7	0.81	0.81	0.81	0
Syracuse, New York	12.0	0.84	0.72	0.85	0.13
San Antonio, Texas	48.6	0.86	0.72	1.00	0.28
Albuquerque, New Mexico	38.3	0.97	0.87	1.04	0.17
Bakersfield, California	35.8	1.01	0.93	1.06	0.13
Phoenix, Arizona^b^	55.1	1.04	0.95	1.15	0.20
Wichita, Kansas	25.3	1.43	1.13	1.55	0.42
Lincoln, Nebraska	35.2	1.57	1.35	1.70	0.35
Salt Lake City, Utah	12.7	1.64	1.28	1.69	0.41

^a^ Selected regions had a large geographic area, an urban center with a population of 100 000 or more, and an observed to expected ratio at the low, middle, or high end of the distribution.

^b^ Includes the Phoenix, Mesa, and Scottsdale metropolitan areas.

**Figure 3.**
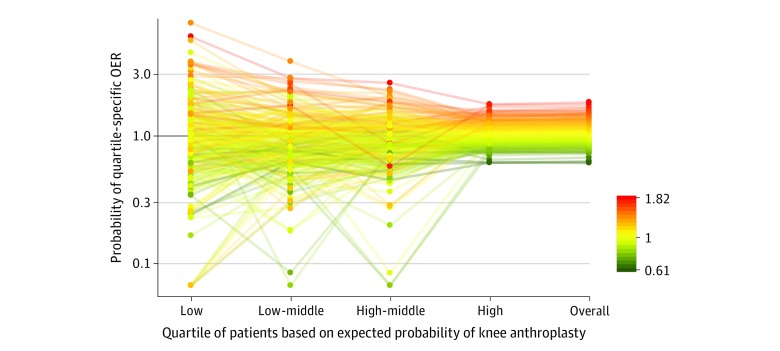
Observed to Expected Ratios (OERs) for Rates of Primary Total Knee Arthroplasty (TKA) in Each Health Referral Region Among White Medicare Beneficiaries by Expected Probability of TKA Expected probabilities were stratified into quartiles from very low (on the left) to highest (on the right), and quartile-specific OERs were computed for each region. Each line represents a health referral region color coded to its overall OER. Health referral regions with very low overall OERs (green) had low OERs regardless of the absolute probability of TKA among beneficiaries (low vs low-middle vs high-middle vs high; ie, green is predominantly below 1.0 in all 4 groups). By contrast, health referral regions with high overall OERs (orange/red) had higher OERs regardless of the absolute probability of TKA (ie, orange/red is predominantly above 1.5 in all 4 groups). These patterns indicate that the overall OER is associated with a region across beneficiaries with a range of likelihoods of TKA.

**Figure 4.**
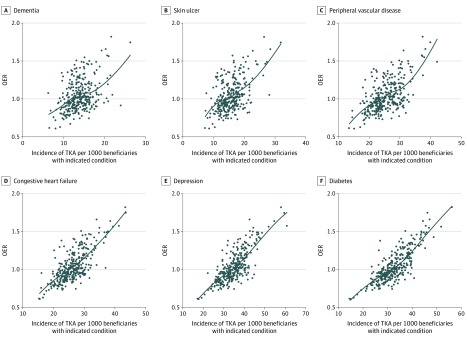
Associations Between Rates of Primary Total Knee Arthroplasty (TKA) Among White Medicare Beneficiaries With Different Comorbid Conditions and the Observed to Expected Ratio (OER) for Rates of TKA by Health Referral Region Each black dot represents a health referral region; black lines, locally estimated scatterplot smoothing curves of the association.

The OERs were stable across subsets of beneficiaries with either low or high estimated probabilities of TKA (Figure 3). This pattern indicated that a broader spectrum of beneficiaries received TKA in high OER regions, whereas physicians and patients in low OER regions were more discriminating. Consistent with this finding, rates of TKA among beneficiaries with dementia (*r* = 0.36; 95% CI, 0.25-0.46), peripheral vascular disease (*r* = 0.52; 95% CI, 0.42-0.61), and chronic skin ulcers (*r* = 0.43; 95% CI, 0.32-0.53) were higher in regions with high OERs, as were rates among beneficiaries with congestive heart failure (*r* = 0.67; 95% CI, 0.59-0.74), depression (*r* = 0.70; 95% CI, 0.63-0.77), and diabetes (*r* = 0.75; 95% CI, 0.68-0.81) (Figure 4). Rates of TKA among 65- to 69-year-olds with no comorbidity were also higher in regions with high OERs (*r* = 0.72; 95% CI, 0.65-0.78) (eFigure 5 in the Supplement).

The number of TKA surgeons per HRR ranged from 48 to 1047. The number of TKA procedures per surgeon was similar in regions with low and high OERs (eFigure 6 in the Supplement).

Analyses of rates among black, Hispanic, and Asian beneficiaries were limited by low representation within HRRs. Only 6 HRRs included at least 15 000 Hispanic beneficiaries, which corresponded to the smallest white HRR population. In 42 HRRs that included at least 15 000 black beneficiaries, OERs ranged from 0.71 to 1.2 (eTable 5 in the Supplement). The OERs in black or in white beneficiaries in these regions were positively correlated and generally higher among black beneficiaries (eFigure 7 in the Supplement).

## Discussion

Substantial variation in rates of primary TKA were present across the US despite adjustment for differences in patient characteristics across regions. Many areas extending from the upper Midwest to the Mountain West had higher than expected rates, whereas several large metropolitan areas had lower than expected rates. This pattern is similar to that first described in 1988, indicating enduring regional differences in care.^7^

Ethnic minorities, particularly black Americans, are one-half to one-quarter as likely to have TKA as white Americans, regardless of medical insurance status.^9,10^ Unfamiliarity with TKA, expectations of less benefit, and risk aversion contribute to lower utilization rates.^13^ These perceptual differences may have resulted in low rates in regions with large minority populations, which, together with the uneven distribution of minorities among HRRs, confounded assessment of regional variation in TKA rates. Stratified analyses were needed to remove this bias.

Among white beneficiaries, geographic barriers to outpatient care likely contributed to high OERs in some regions, particularly in the west. Total knee arthroplasty may be used as a substitute for ongoing conservative care in places where travel to visits is difficult, particularly during winter, and where there are fewer primary care physicians, rehabilitation specialists, and rheumatologists.^39^ By contrast, conservative care may be continued longer in regions with greater proximity to clinicians. This substitution may explain the counterintuitive finding of higher rates of TKA and other surgical procedures in rural areas.^27,40^ Higher than expected rates in the urban centers of these regions, however, suggested that geographic barriers to care do not fully account for the high OERs.

Regions with high OERs tended to be associated with more TKA surgeons per capita than regions with lower OERs. Two previous studies^7,16^ did not find associations with the supply of orthopedic surgeons, perhaps because these studies counted all orthopedic surgeons and not only those who performed TKA, or because they did not examine OERs or perform race/ethnicity–specific analyses. More importantly, beneficiaries likely to have TKA differed across regions. Beneficiaries in high OER regions were more likely to have TKA irrespective of their estimated probability of TKA. Rates among beneficiaries with relative contraindications to TKA or with conditions associated with poorer outcomes were higher in regions with high OERs.^30^ Rates were also higher among younger beneficiaries at the opposite end of the risk spectrum. Together, these results suggested that surgeons in high OER regions appeared less discriminating in the types of patients recommended for TKA than surgeons in other regions. Higher rates among beneficiaries with relative contraindications suggested the overuse of TKA in some high OER regions rather than underuse of TKA in regions with OERs close to 1.0. A similar approach could be applied to investigate overuse and underuse of other procedures.

Twenty HRRs had OERs less than 0.80 among white beneficiaries, including major metropolitan areas such as New York City and Chicago. This may reflect longer reliance on conservative treatments or less handicap associated with knee arthritis among these beneficiaries. However, it may also represent some degree of surgical undercapacity. Boundary-crossing by beneficiaries from nearby regions may also increase competition for allotments that would otherwise be available to city residents. Regions with lower OERs among white beneficiaries also had lower OERs among black beneficiaries, indicating that rates in these groups were not compensatory.

### Limitations

Our study was limited in that most analyses were restricted to white beneficiaries. We modeled expected rates based on clinical characteristics associated with TKA, but because this procedure is discretionary, substantial variation in the use of TKA is expected even among patients with similar medical histories. We did not have measures of joint pain or function, so we could not assess the appropriateness of TKA based on these measures. However, we are not aware of evidence indicating that knee symptom severity varies by region. We also did not have firsthand data from surgeons or beneficiaries regarding their propensity for TKA. Prior surveys have reported that physician enthusiasm for TKA or spinal surgery is the main predictor of small-area variations in surgical procedure rates.^14,41^ Finally, variability among patients within HRRs may obscure differences among HRRs. However, smaller levels of aggregation would likely not provide stable TKA estimates.

## Conclusions

Our results identified factors beyond patient characteristics that may be associated with regional variations in TKA rates. First, among beneficiaries who live outside urban centers, particularly in the Midwest and West, TKA was likely used as a more definitive treatment of knee arthritis in place of ongoing outpatient management. Second, decision-making surrounding TKA differed among regions, with generally lower thresholds for surgery in regions with higher than expected rates. The associations among patients with relative contraindications suggested overuse of TKA in some of the high OER regions. Our data cannot determine what proportion of this threshold variation was due to surgeon practice style or to patient preference. However, the consistent pattern of association across patient subgroups regardless of comorbidity suggested that this variation was not likely solely due to patient preference.

## Notes

Supplement. **eTable 1.** Comorbid Conditions **eTable 2.** Race/Ethnicity Composition of the Ten Health Referral Regions With the Lowest and Highest Observed/Expected Ratios for Rates of Primary Total Knee Arthroplasty **eTable 3.** Distribution of Demographic and Clinical Characteristics of White Medicare Beneficiaries Age 65 to 89 Among Health Referral Regions **eTable 4.** Results of the Poisson Regression Model for Expected Rates of Primary Total Knee Arthroplasty Among White Medicare Beneficiaries in 2013 **eTable 5.** Observed/Expected Ratios for Rates of Primary Total Knee Arthroplasty Among White or Black Medicare Beneficiaries by Health Referral Region **eFigure 1.** Observed/Expected Ratios for Rates of Primary Total Knee Arthroplasty Among Medicare Beneficiaries Age 65 to 89 in 2011-2015, by Health Referral Region **eFigure 2.** Association Between Observed/Expected Ratios for Rates of Primary Total Knee Arthroplasty by Health Referral Region and the Percent of White, Black, Hispanic, and Asian Medicare Beneficiaries Living in the Health Referral Region **eFigure 3.** Distribution of Observed/Expected Ratios for Rates of Primary Total Knee Arthroplasty Among White Medicare Beneficiaries With Expected Rates Based on the Age- and Sex-Adjusted Model, or Based on the Full Model **eFigure 4.** Range of Observed/Expected Ratios Over the 5 Study Years in Each Health Referral Region **eFigure 5.** Association Between Rates of Primary Total Knee Arthroplasty Among White Medicare Beneficiaries Who Were Age 65 to 69 With No Comorbidity and the Observed/Expected Ratio for Rates of Total Knee Arthroplasty by Health Referral Region **eFigure 6.** Percentage of Primary Total Knee Arthroplasties Among White Medicare Beneficiaries by High Volume and Low Volume Surgeons in Each Health Referral Region **eFigure 7.** Association Between the Observed/Expected Ratios for Rates of Primary Total Knee Arthroplasty Between Black and White Medicare Beneficiaries
