# Supplementary material for: Regional Variation in Rates of Total Knee Arthroplasty Among Medicare Beneficiaries
Source: JAMA Netw Open. 2020 Apr 28;3(4):e203717. doi: 10.1001/jamanetworkopen.2020.3717 (PMC7189226; doi:10.1001/jamanetworkopen.2020.3717)
Supplement: Supplement. — eTable 1. Comorbid Conditions eTable 2. Race/Ethnicity Composition of the Ten Health Referral Regions With the Lowest and Highest Observed/Expected Ratios for Rates of Primary Total Knee Arthroplasty eTable 3. Distribution of Demographic and Clinical Characteristics of White Medicare Beneficiaries Age 65 to 89 Among Health Referral Regions eTable 4. Results of the Poisson Regression Model for Expected Rates of Primary Total Knee Arthroplasty Among White Medicare Beneficiaries in 2013 eTable 5. Observed/Expected Ratios for Rates of Primary Total Knee Arthroplasty Among White or Black Medicare Beneficiaries by Health Referral Region eFigure 1. Observed/Expected Ratios for Rates of Primary Total Knee Arthroplasty Among Medicare Beneficiaries Age 65 to 89 in 2011-2015, by Health Referral Region eFigure 2. Association Between Observed/Expected Ratios for Rates of Primary Total Knee Arthroplasty by Health Referral Region and the Percent of White, Black, Hispanic, and Asian Medicare Beneficiaries Living in the Health Referral Region eFigure 3. Distribution of Observed/Expected Ratios for Rates of Primary Total Knee Arthroplasty Among White Medicare Beneficiaries With Expected Rates Based on the Age- and Sex-Adjusted Model, or Based on the Full Model eFigure 4. Range of Observed/Expected Ratios Over the 5 Study Years in Each Health Referral Region eFigure 5. Association Between Rates of Primary Total Knee Arthroplasty Among White Medicare Beneficiaries Who Were Age 65 to 69 With No Comorbidity and the Observed/Expected Ratio for Rates of Total Knee Arthroplasty by Health Referral Region eFigure 6. Percentage of Primary Total Knee Arthroplasties Among White Medicare Beneficiaries by High Volume and Low Volume Surgeons in Each Health Referral Region eFigure 7. Association Between the Observed/Expected Ratios for Rates of Primary Total Knee Arthroplasty Between Black and White Medicare Beneficiaries [file jamanetwopen-3-e203717-s001.pdf]

Supplementary Online Content

Ward MM, Dasgupta A. Regional variation in rates of total knee arthroplasty among Medicare beneficiaries. *JAMA Netw Open*. 2020;3(4):e203717. doi:10.1001/jamanetworkopen.2020.3717

eTable 1. Comorbid Conditions
eTable 2. Race/Ethnicity Composition of the Ten Health Referral Regions With the Lowest and Highest Observed/Expected Ratios for Rates of Primary Total Knee Arthroplasty
eTable 3. Distribution of Demographic and Clinical Characteristics of White Medicare Beneficiaries Age 65 to 89 Among Health Referral Regions
eTable 4. Results of the Poisson Regression Model for Expected Rates of Primary Total Knee Arthroplasty Among White Medicare Beneficiaries in 2013
eTable 5. Observed/Expected Ratios for Rates of Primary Total Knee Arthroplasty Among White or Black Medicare Beneficiaries by Health Referral Region
eFigure 1. Observed/Expected Ratios for Rates of Primary Total Knee Arthroplasty Among Medicare Beneficiaries Age 65 to 89 in 2011-2015, by Health Referral Region
eFigure 2. Association Between Observed/Expected Ratios for Rates of Primary Total Knee Arthroplasty by Health Referral Region and the Percent of White, Black, Hispanic, and Asian Medicare Beneficiaries Living in the Health Referral Region
eFigure 3. Distribution of Observed/Expected Ratios for Rates of Primary Total Knee Arthroplasty Among White Medicare Beneficiaries With Expected Rates Based on the Age- and Sex-Adjusted Model, or Based on the Full Model
eFigure 4. Range of Observed/Expected Ratios Over the 5 Study Years in Each Health Referral Region
eFigure 5. Association Between Rates of Primary Total Knee Arthroplasty Among White Medicare Beneficiaries Who Were Age 65 to 69 With No Comorbidity and the Observed/Expected Ratio for Rates of Total Knee Arthroplasty by Health Referral Region
eFigure 6. Percentage of Primary Total Knee Arthroplasties Among White Medicare Beneficiaries by High Volume and Low Volume Surgeons in Each Health Referral Region
eFigure 7. Association Between the Observed/Expected Ratios for Rates of Primary Total Knee Arthroplasty Between Black and White Medicare Beneficiaries

This supplementary material has been provided by the authors to give readers additional information about their work.

© 2020 Ward MM et al. *JAMA Network Open*.

eTable 1. Comorbid Conditions. Definitions were those of the Centers for Medicare and Medicaid Services Chronic Conditions Warehouse. "Ever" conditions were those present at any time from enrollment in Medicare to either the time of total knee arthroplasty (TKA) or the end of each calendar year (for those without TKA). "Recent" conditions were those present in the one year prior to TKA or any time in the calendar year (for those without TKA).

Acute myocardial infarction, recent	Ischemic heart disease, ever
Atrial fibrillation, recent	Congestive heart failure, ever
Chronic skin ulcer, recent	Stroke, ever
Diabetes mellitus, recent	Peripheral vascular disease, ever
Chronic kidney disease, recent	Chronic obstructive pulmonary disease, ever
Depression, recent	Cirrhosis/chronic liver disease, ever
	HIV/AIDS, ever
	Dementia, ever
	Breast cancer, ever
	Lung cancer, ever
	Colorectal cancer, ever
	Endometrial cancer, ever
	Prostate cancer, ever
	Hematological malignancy, ever

© 2020 Ward MM et al. *JAMA Network Open*.

eTable 2. Race/Ethnicity Composition of the Ten Health Referral Regions With the Lowest and Highest Observed/Expected Ratios for Rates of Primary Total Knee Arthroplasty.

Percentages may not add to 100% due to rounding.

Health Referral Region	Observed/ expected ratio	% White	% Black	% Hispanic	% Asian	% Other
Bronx NY	0.52	47.0	33.1	11.7	2.7	5.4
Newark NJ	0.53	62.7	23.9	4.7	3.7	4.9
New York NY	0.54	66.8	16.6	4.1	5.8	6.5
Miami FL	0.65	62.6	9.9	22.9	0.8	3.5
Hackensack NJ	0.67	79.0	5.3	5.4	4.8	5.4
New Brunswick NJ	0.68	78.8	6.4	2.0	6.6	6.0
San Bernardino CA	0.69	70.3	8.1	9.6	6.1	5.7
Honolulu HI	0.70	33.3	0.8	0.3	25.9	39.4
East Long Island NY	0.70	77.8	9.3	2.3	4.8	5.5
Chicago IL	0.70	51.6	35.2	5.1	4.1	3.9
Mason City IA	1.41	98.5	0.1	0.1	0.1	1.0
Wichita KS	1.42	94.0	2.2	1.1	0.8	1.8
Bismarck ND	1.43	95.5	0.1	0.0	0.1	4.2
Sioux Falls SD	1.45	96.5	0.2	0.1	0.2	2.9
Salt Lake City UT	1.48	93.7	0.3	1.4	1.1	3.3
Sioux City IA	1.54	95.2	0.3	1.0	0.7	2.6
Lincoln NE	1.57	96.9	0.5	0.4	0.6	1.4
Ogden UT	1.60	94.3	0.9	1.6	0.9	2.2
Provo UT	1.64	95.5	0.1	2.0	0.5	1.7
Idaho Falls ID	1.72	95.4	0.1	1.5	0.3	2.5

© 2020 Ward MM et al. *JAMA Network Open*.

eTable 3. Distribution of Demographic and Clinical Characteristics of White Medicare Beneficiaries Age 65 to 89 Among Health Referral Regions. Values are percentages, except age, area-based socioeconomic score, and annual number of outpatient visits.

Variable	Minimum	10%	Median	90%	Maximum
Mean age, years	72.3	73.4	74.0	74.6	75.1
Men	42.7	44.3	45.7	48.0	52.2
Acute myocardial infarction	0.3	0.5	0.7	1.0	1.5
Atrial fibrillation	5.0	6.8	8.3	9.5	10.6
Breast cancer	2.9	3.8	4.3	4.9	5.9
Congestive heart failure	12.0	15.7	20.3	25.4	34.1
Chronic kidney disease	6.3	10.7	13.2	15.8	22.1
Colorectal cancer	1.4	1.8	2.2	2.6	3.1
Chronic obstructive pulmonary disease	11.0	16.0	21.9	27.8	35.2
Dementia	6.0	8.1	10.4	12.5	18.6
Depression	6.9	9.9	11.9	13.7	16.3
Diabetes mellitus	11.2	16.7	21.6	25.5	34.2
Endometrial cancer	0.3	0.5	0.7	0.9	1.2
Hematological malignancy	1.2	1.5	1.7	2.0	2.7
HIV/AIDS	0.0	0.0	0.1	0.1	1.0
Ischemic heart disease	24.1	29.9	39.1	49.3	59.6
Cirrhosis/chronic liver disease	3.2	4.5	5.7	7.7	17.6
Lung cancer	0.3	0.8	1.1	1.4	1.8
Prostate cancer	3.2	3.9	4.6	5.5	7.2
Peripheral vascular disease	6.7	11.0	16.3	22.0	30.6
Chronic skin ulcer	1.7	2.7	3.4	4.4	5.9
Stroke	7.0	8.9	11.9	14.2	17.0
Annual number of outpatient visits	3.6	5.3	6.8	8.5	11.6
Any knee osteoarthritis visit	3.8	5.4	6.8	8.0	10.5
Knee symptoms	3.3	4.9	5.9	7.5	9.6

© 2020 Ward MM et al. *JAMA Network Open*.

Variable	Minimum	10%	Median	90%	Maximum
Knee osteoarthritis or knee symptoms	5.9	8.4	9.7	11.2	12.7
Percent obese	23.5	30.9	36.5	40.4	47.3
Percent with physical occupation	8.3	12.0	14.8	18.3	26.8
Percent smokers	9.0	17.1	22.3	26.4	29.8
Percent Medicare managed care	0.4	10.9	23.6	42.6	59.3
Poor	2.1	4.8	8.0	13.4	35.7
Mean area-based socioeconomic score[a]	-6.8	-2.2	0.9	6.1	13.0
Rural residence	0.0	1.2	24.1	50.3	84.7

[a] Based on seven economic measures of the zip code of residence in the 2010 U.S. Census. A zip code at the national average on all measures would have a score of 0. A zip code whose values on all seven measures were one standard deviation below the national average would have a score of -7.0, and a zip code whose values on all seven measures were one standard deviation above the national average would have a score of +7.0.

© 2020 Ward MM et al. *JAMA Network Open*.

eTable 4. Results of the Poisson Regression Model for Expected Rates of Primary Total Knee Arthroplasty Among White Medicare Beneficiaries in 2013. Results for other years were very similar.

Variable	Estimate	Std. Err.
Intercept	-3.28	0.03
Age: 65-69	0.59	0.02
Age: 70-74	0.65	0.02
Age: 75-79	0.66	0.02
Age: 80-84	0.46	0.02
Age: 85-89 (ref)		
Female	-0.20	0.02
Male (ref)		
Female & Age: 65-69	0.17	0.02
Female & Age: 70-74	0.14	0.02
Female & Age: 75-79	0.11	0.02
Female & Age: 80-84	0.06	0.02
Female & Age: 85-89 (ref)		
Knee osteoarthritis or symptoms	4.70	0.01
Percent obese	0.30	0.09
Percent with physical occupation	0.74	0.09
Percent smokers	0.99	0.09
Acute myocardial infarction	-0.15	0.03
Atrial fibrillation	0.19	0.01
Breast cancer	-0.00	0.01
Congestive heart failure	-0.16	0.01
Chronic kidney disease	0.16	0.01
Colorectal cancer	-0.07	0.02
Chronic obstructive pulmonary disease	-0.12	0.01

© 2020 Ward MM et al. *JAMA Network Open*.

Variable	Estimate	Std. Err.
Dementia	-0.40	0.01
Depression	0.47	0.01
Diabetes mellitus	-0.04	0.01
Endometrial cancer	0.00	0.02
Hematological malignancy	-0.13	0.02
HIV/AIDS	-0.24	0.08
Ischemic heart disease	0.03	0.00
Cirrhosis/chronic liver disease	-0.14	0.01
Lung cancer	-0.29	0.03
Prostate cancer	-0.00	0.01
Peripheral vascular disease	-0.17	0.01
Chronic skin ulcer	-0.18	0.01
Stroke	-0.21	0.01
Poor	-0.55	0.01
Area-based socioeconomic score	-0.01	0.00

© 2020 Ward MM et al. *JAMA Network Open*.

eTable 5. Observed/Expected Ratios for Rates of Primary Total Knee Arthroplasty Among White or Black Medicare Beneficiaries by Health Referral Region. Expected rates were based on race-specific full models. Models for blacks were limited to regions with at least 15,000 black beneficiaries.

Health Referral Region	White	Black
Newark NJ	0.61	0.71
Bronx NY	0.62	0.79
New York NY	0.63	0.88
Miami FL	0.67	0.90
New Orleans LA	0.72	
New Brunswick NJ	0.73	
Fort Lauderdale FL	0.73	0.96
Salisbury MD	0.75	
Hudson FL	0.75	
Hackensack NJ	0.75	
Tupelo MS	0.75	
Jackson TN	0.75	
Kingsport TN	0.76	
Huntington WV	0.78	
East Long Island NY	0.78	0.91
Macon GA	0.78	0.89
Chicago IL	0.78	0.81
Clearwater FL	0.79	
Memphis TN	0.79	0.82
Metairie LA	0.79	
Las Vegas NV	0.80	
Paterson NJ	0.80	
Hickory NC	0.80	
Santa Cruz CA	0.80	
Albany GA	0.80	
Johnson City TN	0.81	
Shreveport LA	0.81	0.80
Lexington KY	0.81	
Tuscaloosa AL	0.82	
Scranton PA	0.82	

© 2020 Ward MM et al. *JAMA Network Open*.

Health Referral Region	White	Black
Bradenton FL	0.83	
Baton Rouge LA	0.83	0.87
Tampa FL	0.83	
Lakeland FL	0.83	
Honolulu HI	0.84	
Ridgewood NJ	0.84	
Syracuse NY	0.84	
Dothan AL	0.84	
Slidell LA	0.84	
Hattiesburg MS	0.84	
Rome GA	0.84	
Los Angeles CA	0.85	1.12
Oxford MS	0.85	
St Petersburg FL	0.85	
Durham NC	0.85	1.02
San Antonio TX	0.86	
Wilkes-Barre PA	0.86	
Worcester MA	0.86	
Springfield MA	0.86	
Raleigh NC	0.86	1.13
Greenville NC	0.87	1.09
Pensacola FL	0.87	
Spartanburg SC	0.87	
El Paso TX	0.87	
Morristown NJ	0.87	
White Plains NY	0.87	
Camden NJ	0.87	0.95
Gainesville FL	0.87	
Winchester VA	0.88	
Allentown PA	0.88	
Panama City FL	0.88	
San Bernardino CA	0.88	
Philadelphia PA	0.88	0.96
Sarasota FL	0.89	

© 2020 Ward MM et al. *JAMA Network Open*.

Health Referral Region	White	Black
Pueblo CO	0.89	
Tallahassee FL	0.89	
Roanoke VA	0.89	
Fresno CA	0.89	
Monroe LA	0.89	
Danville PA	0.90	
Winston-Salem NC	0.90	
Birmingham AL	0.90	0.96
Ocala FL	0.90	
Meridian MS	0.91	
Palm Springs CA	0.91	
Lynchburg VA	0.91	
Buffalo NY	0.91	
Texarkana AR	0.91	
Takoma Park MD	0.91	1.08
Evanston IL	0.91	
Charleston WV	0.91	
Salinas CA	0.91	
Alexandria LA	0.91	
Morgantown WV	0.92	
McAllen TX	0.92	
Chattanooga TN	0.92	
Lafayette LA	0.92	
Youngstown OH	0.92	
Jackson MS	0.92	1.20
Binghamton NY	0.93	
Jonesboro AR	0.93	
Mobile AL	0.93	
Columbus GA	0.93	
Charleston SC	0.93	1.00
Florence SC	0.93	
Orlando FL	0.93	1.12
Montgomery AL	0.94	
York PA	0.94	

© 2020 Ward MM et al. *JAMA Network Open*.

Health Referral Region	White	Black
Sacramento CA	0.94	
Wilmington NC	0.94	
Atlanta GA	0.94	1.02
Burlington VT	0.94	
Pittsburgh PA	0.95	
Newport News VA	0.95	
Ormond Beach FL	0.95	
Lake Charles LA	0.95	
Hartford CT	0.95	
Waco TX	0.95	
Bridgeport CT	0.95	
Gary IN	0.95	
Asheville NC	0.95	
Elyria OH	0.95	
Harlingen TX	0.95	
Greensboro NC	0.95	
Johnstown PA	0.96	
San Diego CA	0.96	
Ventura CA	0.96	
Cleveland OH	0.96	0.98
Munster IN	0.96	
Sayre PA	0.96	
Temple TX	0.96	
Greenville SC	0.96	
Orange County CA	0.96	
Norfolk VA	0.96	0.98
Albany NY	0.96	
Wilmington DE	0.97	
Boston MA	0.97	1.02
Charlottesville VA	0.97	
New Haven CT	0.97	
Albuquerque NM	0.97	
Alameda County CA	0.97	
Fort Smith AR	0.97	

© 2020 Ward MM et al. *JAMA Network Open*.

Health Referral Region	White	Black
Erie PA	0.97	
Nashville TN	0.97	1.18
Reading PA	0.97	
Rochester NY	0.98	
San Mateo County CA	0.98	
Detroit MI	0.98	0.93
Altoona PA	0.98	
Corpus Christi TX	0.99	
Terre Haute IN	0.99	
Blue Island IL	0.99	1.02
Fort Myers FL	0.99	
Houma LA	0.99	
Jacksonville FL	0.99	1.04
Chico CA	1.00	
Elmira NY	1.00	
Abilene TX	1.00	
Owensboro KY	1.00	
Charlotte NC	1.00	1.13
Melrose Park IL	1.00	
Wausau WI	1.01	
Lancaster PA	1.01	
Bakersfield CA	1.01	
Tyler TX	1.01	
Knoxville TN	1.01	
Modesto CA	1.02	
Flint MI	1.02	
Providence RI	1.02	
Washington DC	1.02	1.04
Houston TX	1.02	0.92
Dallas TX	1.02	0.99
Petoskey MI	1.02	
Canton OH	1.02	
Dearborn MI	1.02	
Richmond VA	1.02	1.06

© 2020 Ward MM et al. *JAMA Network Open*.

Health Referral Region	White	Black
Lawton OK	1.02	
Beaumont TX	1.02	
Louisville KY	1.03	
Joliet IL	1.03	
Toledo OH	1.03	
Tacoma WA	1.03	
Manchester NH	1.03	
Baltimore MD	1.03	1.11
Columbia SC	1.03	1.12
Longview TX	1.03	
Aurora IL	1.03	
Hinsdale IL	1.03	
San Angelo TX	1.03	
Phoenix AZ	1.04	
Huntsville AL	1.04	
Milwaukee WI	1.04	
Medford OR	1.04	
San Francisco CA	1.04	
Everett WA	1.05	
Mesa AZ	1.05	
Fort Worth TX	1.05	
Stockton CA	1.06	
Sun City AZ	1.06	
Contra Costa County CA	1.06	
Gulfport MS	1.06	
Savannah GA	1.06	
Augusta GA	1.06	1.16
Covington KY	1.06	
San Jose CA	1.06	
Santa Barbara CA	1.07	
Rockford IL	1.07	
St Louis MO	1.07	1.11
Redding CA	1.07	
Austin TX	1.08	

© 2020 Ward MM et al. *JAMA Network Open*.

Health Referral Region	White	Black
Muncie IN	1.08	
Salem OR	1.08	
Indianapolis IN	1.08	1.14
Harrisburg PA	1.08	
Cape Girardeau MO	1.08	
Paducah KY	1.08	
Little Rock AR	1.08	1.07
Ann Arbor MI	1.09	
Kettering OH	1.09	
Colorado Springs CO	1.09	
Portland ME	1.09	
Cincinnati OH	1.11	
Akron OH	1.11	
Royal Oak MI	1.11	
Evansville IN	1.11	
Elgin IL	1.11	
Reno NV	1.12	
Springfield MO	1.12	
Odessa TX	1.13	
Columbus OH	1.13	
Marshfield WI	1.14	
Eugene OR	1.14	
Joplin MO	1.14	
Oklahoma City OK	1.15	
Santa Rosa CA	1.15	
Bryan TX	1.15	
Muskegon MI	1.15	
Appleton WI	1.15	
Peoria IL	1.15	
Dayton OH	1.15	
Springdale AR	1.15	
Grand Junction CO	1.16	
Yakima WA	1.16	
St Joseph MI	1.17	

© 2020 Ward MM et al. *JAMA Network Open*.

Health Referral Region	White	Black
Wichita Falls TX	1.18	
Napa CA	1.18	
Tucson AZ	1.19	
Arlington VA	1.19	
Grand Rapids MI	1.19	
Urbana IL	1.19	
Victoria TX	1.20	
Seattle WA	1.20	
Tulsa OK	1.20	
Kansas City MO	1.20	0.96
Bloomington IL	1.21	
Saginaw MI	1.21	
Boulder CO	1.21	
Lansing MI	1.21	
Pontiac MI	1.21	
Madison WI	1.22	
Kalamazoo MI	1.22	
Grand Forks ND	1.22	
Casper WY	1.23	
Columbia MO	1.23	
Portland OR	1.24	
Lebanon NH	1.26	
Fort Wayne IN	1.26	
Amarillo TX	1.26	
Bend OR	1.27	
Lafayette IN	1.28	
San Luis Obispo CA	1.28	
Duluth MN	1.28	
Spokane WA	1.28	
Minot ND	1.28	
South Bend IN	1.29	
Springfield IL	1.30	
Anchorage AK	1.30	
St Paul MN	1.30	

© 2020 Ward MM et al. *JAMA Network Open*.

Health Referral Region	White	Black
Fort Collins CO	1.31	
Waterloo IA	1.31	
Rapid City SD	1.32	
La Crosse WI	1.32	
Davenport IA	1.33	
Fargo Moorhead ND	1.33	
Topeka KS	1.33	
Denver CO	1.34	
Neenah WI	1.35	
Lubbock TX	1.35	
Rochester MN	1.35	
Missoula MT	1.36	
Minneapolis MN	1.36	
Olympia WA	1.37	
Bangor ME	1.38	
Omaha NE	1.38	
St Cloud MN	1.40	
Billings MT	1.40	
Marquette MI	1.41	
Des Moines IA	1.41	
Cedar Rapids IA	1.43	
Wichita KS	1.43	
Green Bay WI	1.45	
Dubuque IA	1.46	
Iowa City IA	1.46	
Great Falls MT	1.47	
Boise ID	1.49	
Mason City IA	1.49	
Greeley CO	1.49	
Bismarck ND	1.50	
Traverse City MI	1.51	
Sioux Falls SD	1.55	
Lincoln NE	1.57	
Ogden UT	1.58	

© 2020 Ward MM et al. *JAMA Network Open*.

Health Referral Region	White	Black
Salt Lake City UT	1.64	
Sioux City IA	1.66	
Provo UT	1.75	
Idaho Falls ID	1.82	

© 2020 Ward MM et al. *JAMA Network Open*.

eFigure 1. Observed/Expected Ratios for Rates of Primary Total Knee Arthroplasty Among Medicare Beneficiaries Age 65 to 89 in 2011-2015, by Health Referral Region.

Expected rates were based on models that adjusted for age, sex, and race/ethnicity.

OER = observed/expected ratio.

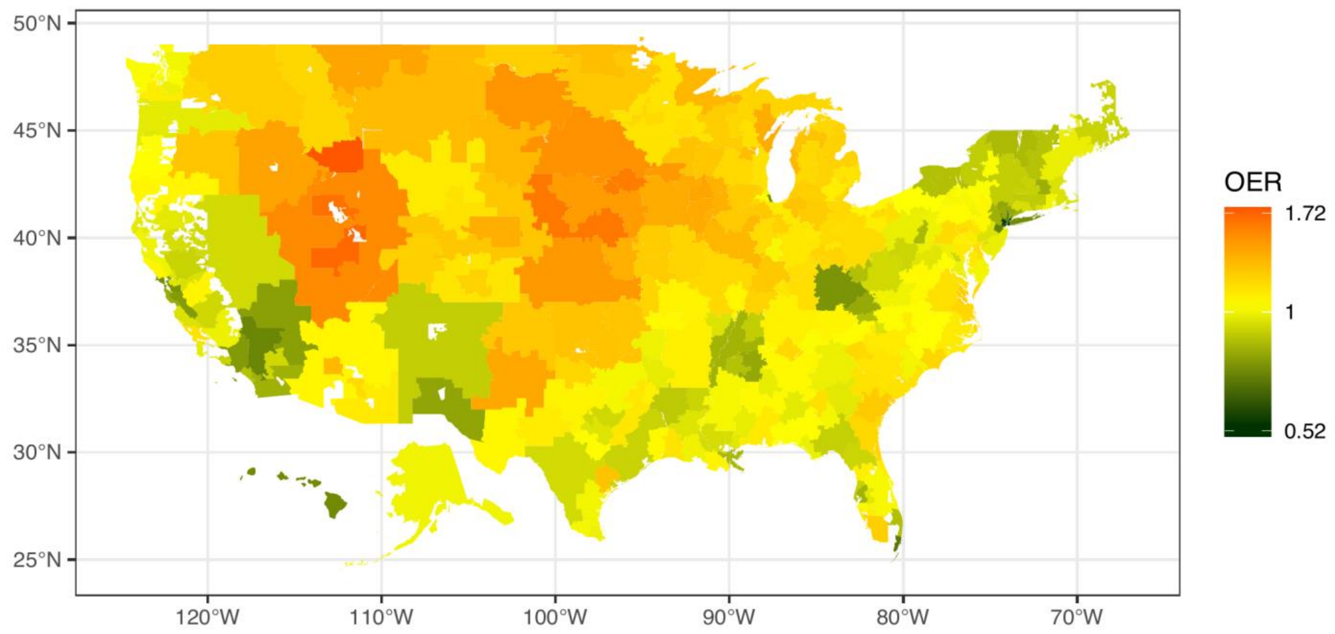

© 2020 Ward MM et al. *JAMA Network Open*.

eFigure 2. Association Between Observed/Expected Ratios for Rates of Primary Total Knee Arthroplasty by Health Referral Region and the Percent of White, Black, Hispanic, and Asian Medicare Beneficiaries Living in the Health Referral Region

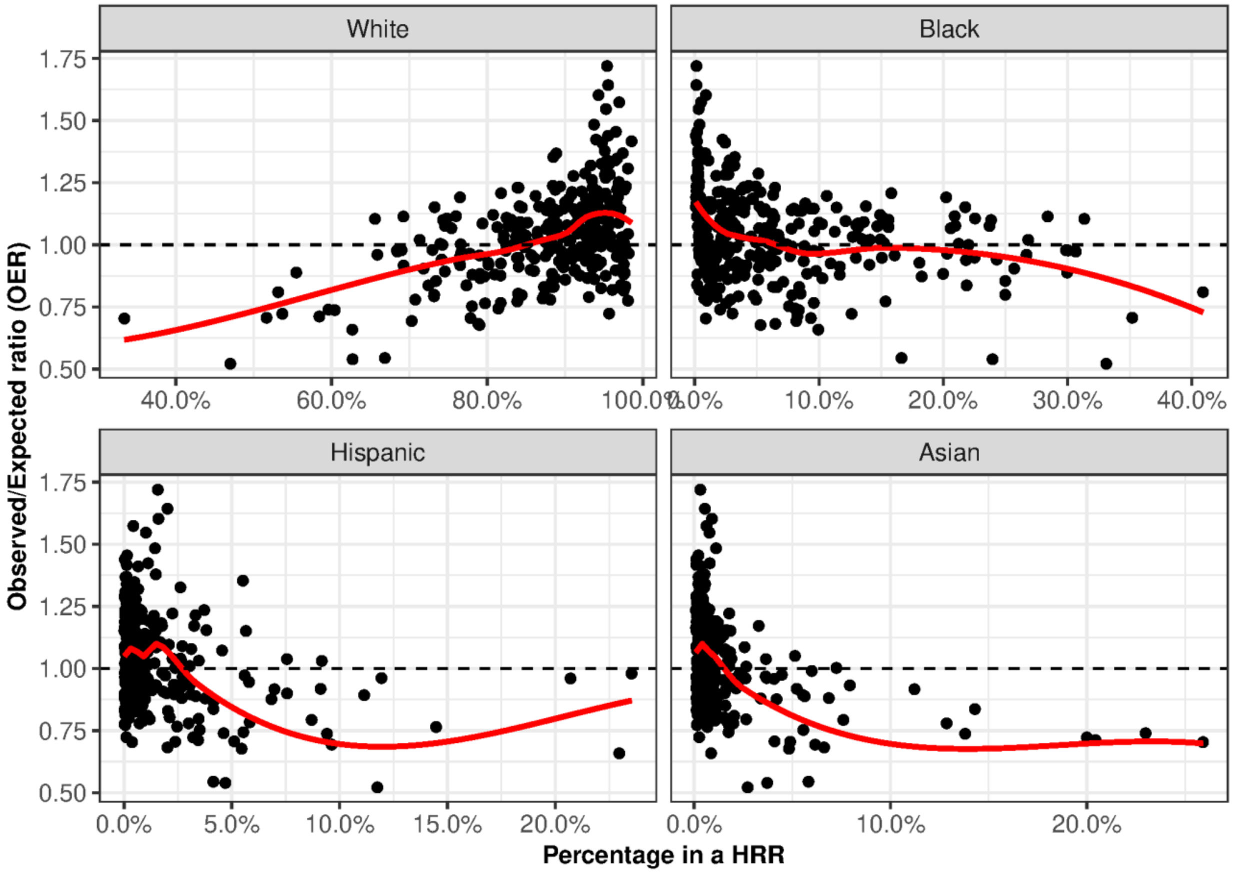

© 2020 Ward MM et al. *JAMA Network Open*.

eFigure 3. Distribution of Observed/Expected Ratios for Rates of Primary Total Knee Arthroplasty Among White Medicare Beneficiaries With Expected Rates Based on the Age- and Sex-Adjusted Model, or Based on the Full Model. The full model adjusted for age, sex, presence of knee symptoms, area-based measures of obesity, smoking, and physically demanding occupations, 20 comorbid conditions, poverty, and area-based socioeconomic score. Observed/expected ratios based on the full model were more concentrated around 1.0 (kurtosis 3.079) than the age-and sex-adjusted model (kurtosis 4.21), indicating a 29% reduction in spread among Health Referral Regions.

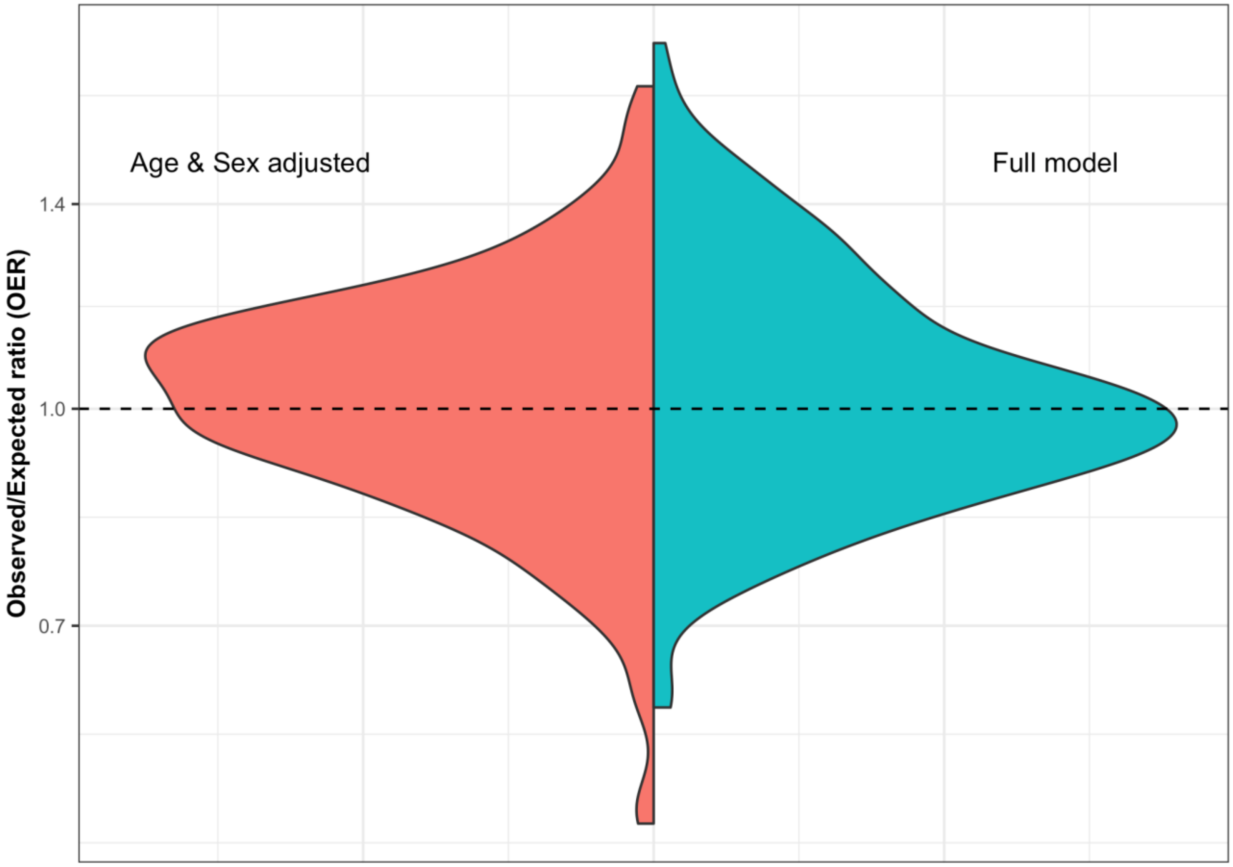

© 2020 Ward MM et al. *JAMA Network Open*.

eFigure 4. Range of Observed/Expected Ratios Over the 5 Study Years in Each Health Referral Region. Error bars represent the minimum and maximum ratio. Observed/expected ratios are ordered by the size of the Medicare population in each region.

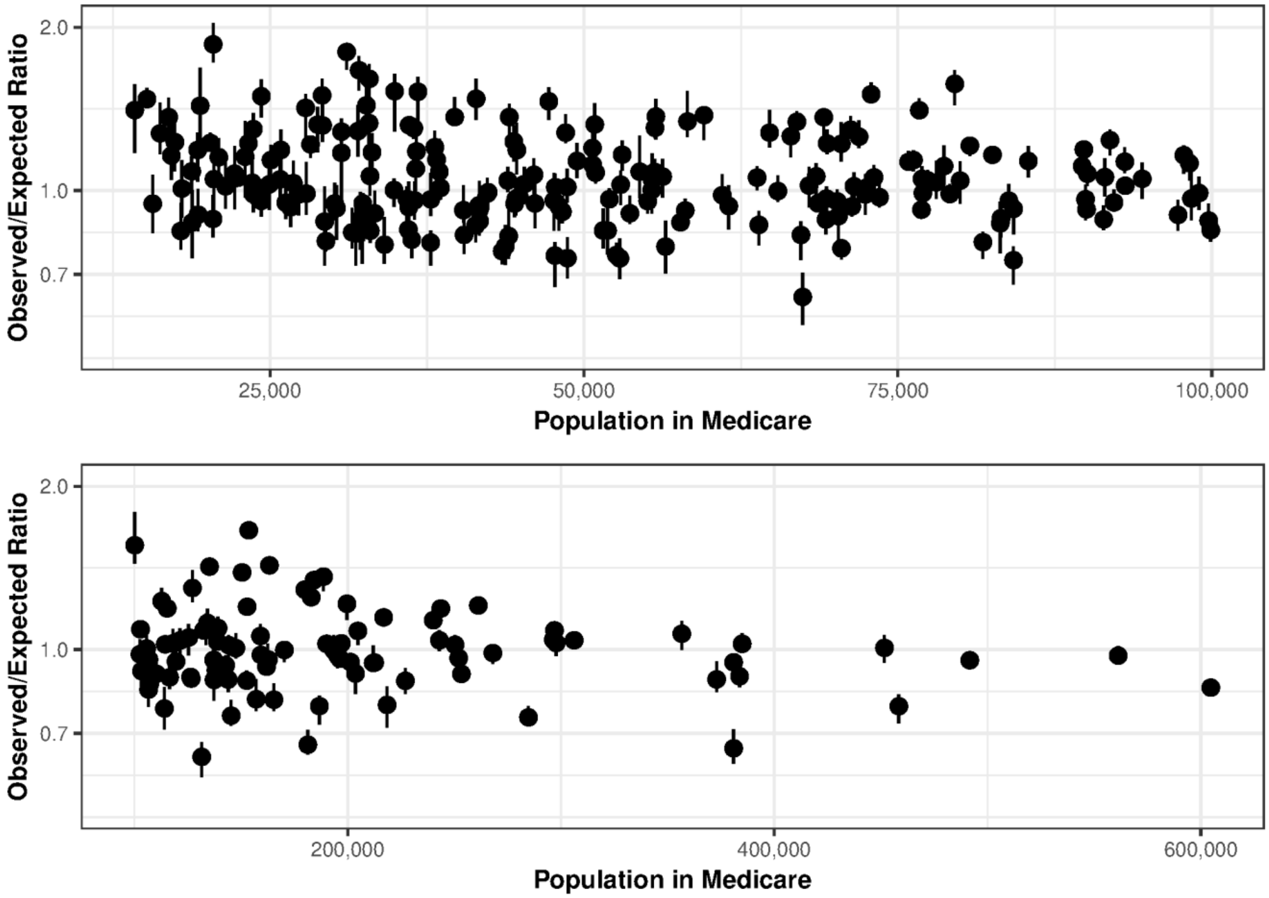

© 2020 Ward MM et al. *JAMA Network Open*.

eFigure 5. Association Between Rates of Primary Total Knee Arthroplasty Among White Medicare Beneficiaries Who Were Age 65 to 69 With No Comorbidity and the Observed/Expected Ratio for Rates of Total Knee Arthroplasty by Health Referral Region

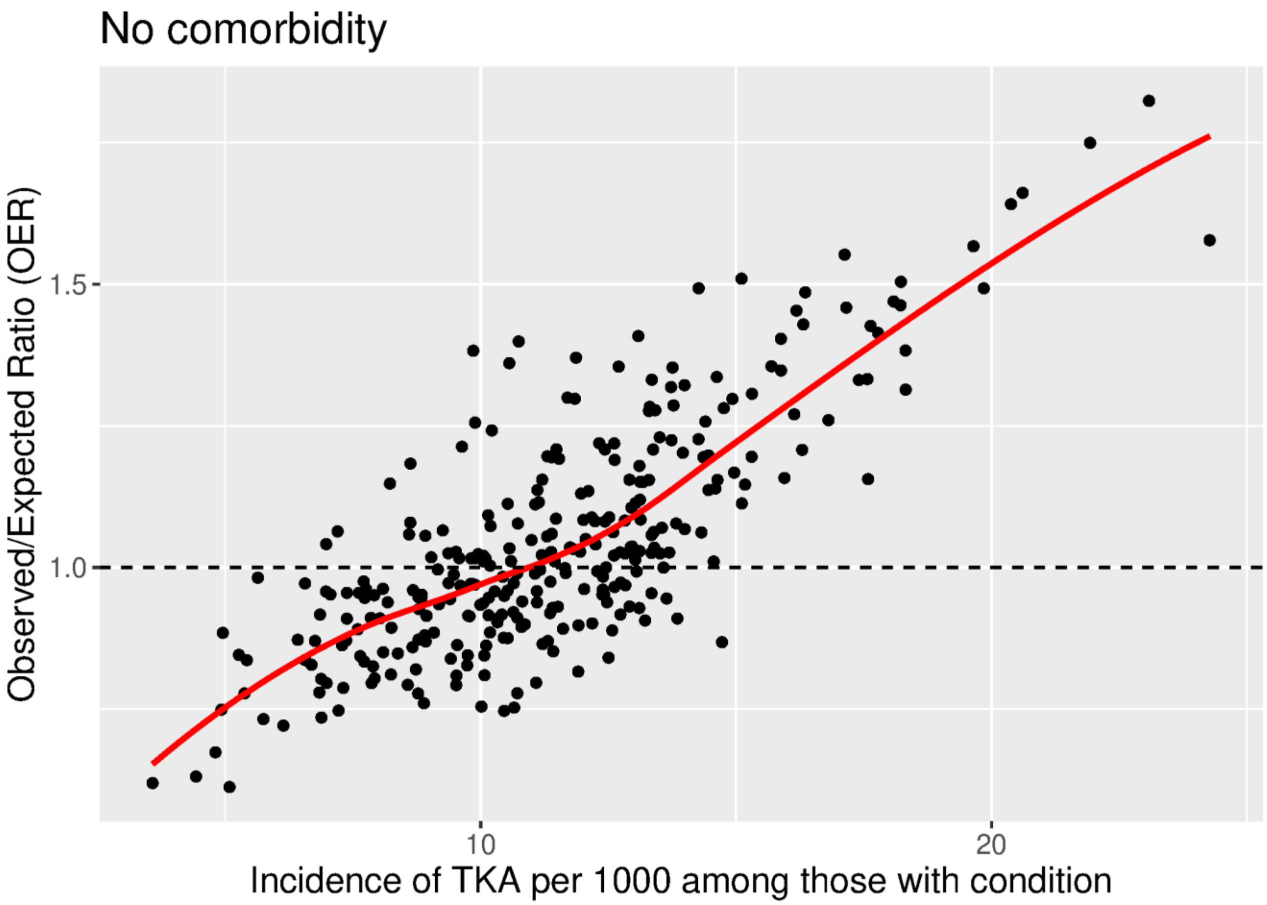

© 2020 Ward MM et al. *JAMA Network Open*.

eFigure 6. Percentage of Primary Total Knee Arthroplasties Among White Medicare Beneficiaries by High Volume and Low Volume Surgeons in Each Health Referral Region. Each column represents a region, ordered from low to high based on their observed/expected ratio. Dark blue represents the percentage of knee arthroplasties in the region performed by surgeons who performed on average 50 or more procedures annually.

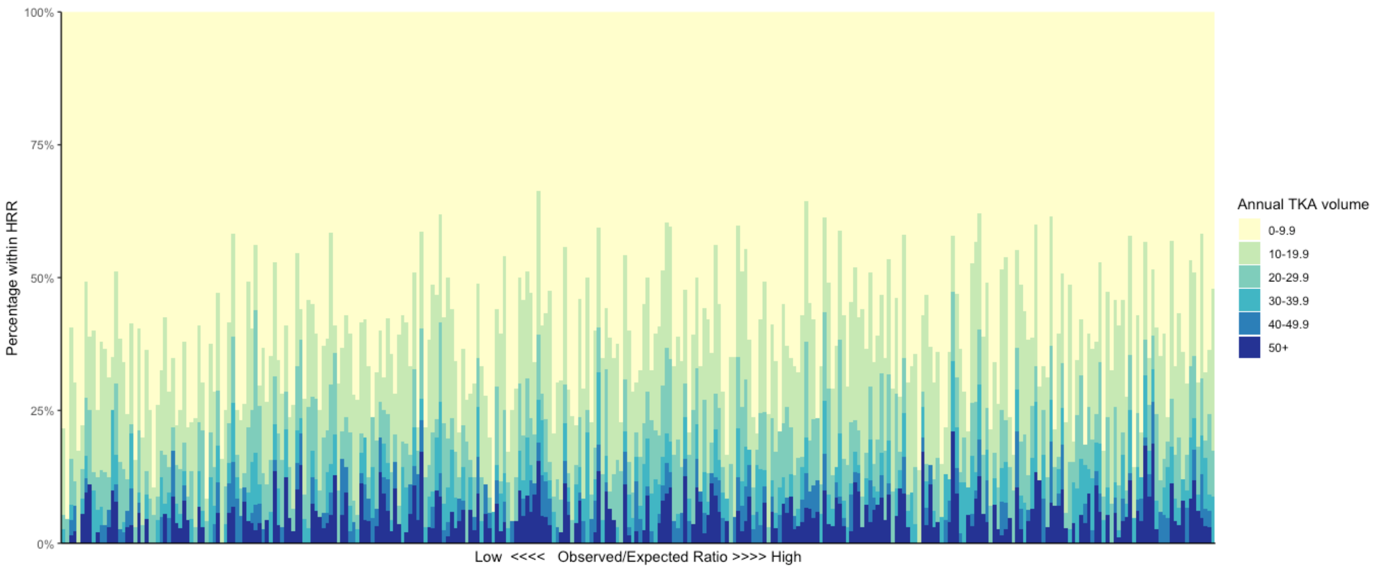

© 2020 Ward MM et al. *JAMA Network Open*.

eFigure 7. Association Between the Observed/Expected Ratios for Rates of Primary Total Knee Arthroplasty Between Black and White Medicare Beneficiaries. Health Referral Regions that included at least 15,000 black beneficiaries are shown.

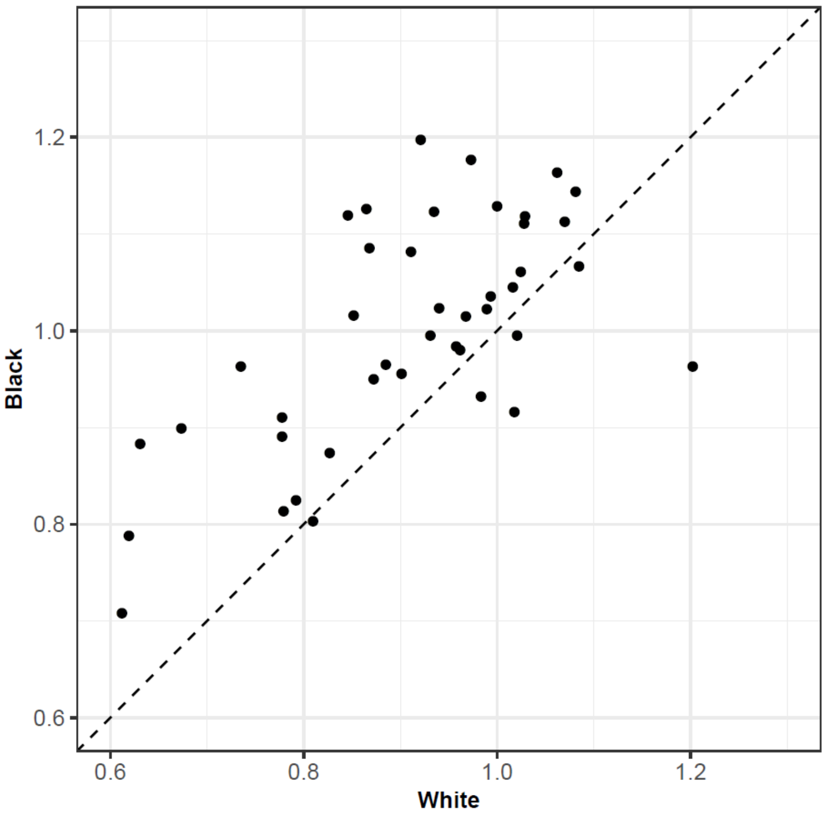

© 2020 Ward MM et al. *JAMA Network Open*.
